# Emodin reverses leukemia multidrug resistance by competitive inhibition and downregulation of P-glycoprotein

**DOI:** 10.1371/journal.pone.0187971

**Published:** 2017-11-09

**Authors:** Hongping Min, Miaomiao Niu, Weilin Zhang, Jia Yan, Jiachang Li, Xiying Tan, Bo Li, Mengxiang Su, Bin Di, Fang Yan

**Affiliations:** 1 Key Laboratory on Protein Chemistry and Structural Biology, China Pharmaceutical University, Nanjing, China; 2 Key Laboratory of Drug Quality Control and Pharmacovigilance (China Pharmaceutical University), Ministry of Education, Nanjing, China; 3 Department of Pharmacology, China Pharmaceutical University, Nanjing, China; 4 Jiangsu Province Hospital of Chinese Traditional Medicine, Nanjing, China; Columbia University, UNITED STATES

## Abstract

Development of multidrug resistance (MDR) is a continuous clinical challenge partially due to the overexpression of P-glycoprotein (P-gp) for chronic myelogenous leukemia (CML) patients. Herein, we evaluated the inhibitory potency of emodin, a natural anthraquinone derivative isolated from *Rheum palmatum* L, on P-gp in P-gp positive K562/ADM cells. Competition experiments combined with molecular docking analysis were utilized to investigate the binding modes between emodin and binding sites of P-gp. Emodin reversed adriamycin resistance in K562/ADM cells accompanied with the decrease of P-gp protein expression, further increasing the uptake of rhodamine123 in both K562/ADM and Caco-2 cells, indicating the inhibition of P-gp efflux function. Moreover, when incubated with emodin under different conditions where P-gp was inhibited, K562/ADM cells displayed increasing intracellular uptake of emodin, suggesting that emodin may be the potential substrate of P-gp. Importantly, rhodamine 123 could increase the *K*_*intrinsic*_ (*K*_*i*_) value of emodin linearly, whereas, verapamil could not, implying that emodin competitively bound to the R site of P-gp and noncompetition existed between emodin and verapamil at the M site, in a good accordance with the results of molecular docking that emodin bound to the R site of P-gp with higher affinity. Based on our results, we suggest that emodin might be used to modulate P-gp function and expression.

## Introduction

Chronic myelogenous leukemia (CML) results from the neoplastic transformation of haematopoietic stem cell. The hallmark genetic abnormality of CML is a t(9;22)(q34;q11) translocation named the "Philadelphia chromosome", which generates the BCR–ABL fusion gene [1, 2]. In the past decades, development of specific TKIs such as imatinib (IM) revolutionized the treatment of CML, however, a significant number of patients develop drug resistance, especially in late stages of the disease. The drug resistance mechanisms involve many factors, which are mainly divided into Bcr/abl-dependent and Bcr/abl-independent mechanisms [3]. Among the latter is the MDR phenotype which is mostly associated with the overexpression of P-glycoprotein (P-gp) [4]. P-gp, an ATP-binding cassette (ABC) membrane transporter encoded by multidrug resistance 1 (MDR1), is commonly located at the plasma membrane and has been demonstrated to function as an ATP-dependent efflux pump for diverse naturally occurring hydrophobic anticancer drugs such as adriamycin [5]. As a consequence of this, many anticancer regents could not accumulate in tumor cells efficiently to reach the sufficient therapeutic concentrations and led to MDR [6]. Therefore, it is necessary to develop P-gp inhibitors to modulate its pumping activity so as to circumvent P-gp mediated MDR in CML.

**Fig 1:**
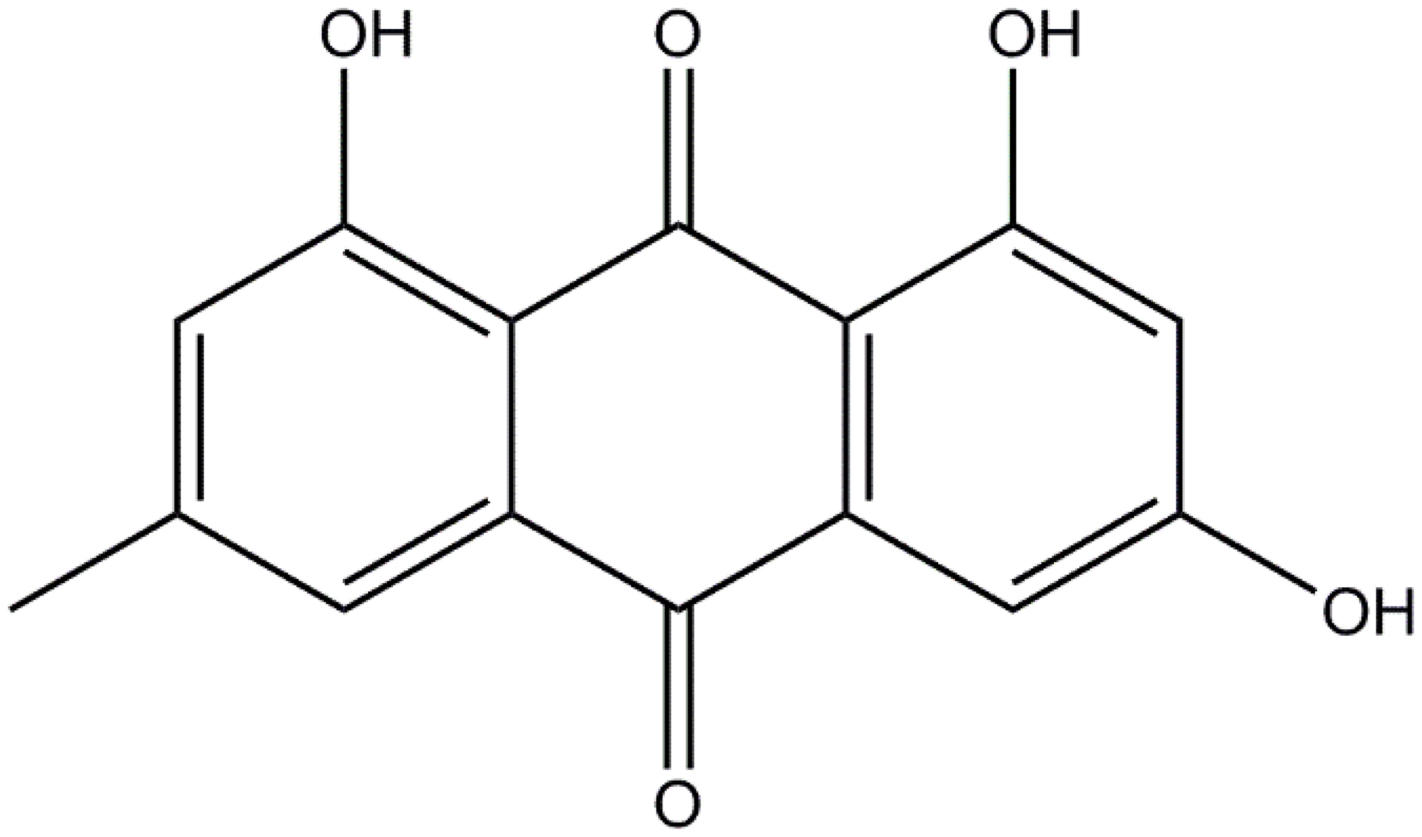
Chemical structure of emodin (1,3,8-trihydroxy-6-methylanthraquinone).

Emodin (1, 3, 8-trihydroxy-6-methylanthra-quinone) (Fig 1), a natural anthraquinone derivative isolated from *Rheum palmatum* L., has been reported to potentiate the anti-proliferation of various chemotherapeutic agents [7]. It is demonstrated that emodin reverses MDR in resistant HL-60/ADR cells [8], and emodin and AZT exhibit synergistic growth-inhibitory effects in K562/ADM cells [9]. In addition, emodin is confirmed to be the inhibitor of P-gp by inhibiting P-gp efflux function and protein expression in Caco-2 cells [10]. Subsequently, it is proposed that emodin is the potential substrate of P-gp and further hypothesized that emodin inhibits the P-gp function by competitively binding to the transport sites [11]. However, it is unreported whether emodin could reverse the adriamycin resistance in K562/ADM cells. Moreover, whether emodin is the inhibitor and substrate of P-gp is controversial in different experimental conditions [11, 12]. Importantly, it has to be noted that the hypothesis about the binding modes of emodin on P-gp by Li et al is only based on the molecular docking analysis without accurate experimental data, and to date, the definite binding sites of emodin on P-gp are not clear yet. Hence, more direct experimental data and more detailed molecular docking analysis are urgently needed to illuminate the binding sites of emodin on P-gp.

In this paper, we investigated the reversal effects of emodin in adriamycin resistant K562/ADM cells and its inhibitory effects on P-gp protein expression as well as its efflux function. Serial studies were performed aiming to clarify whether emodin is the substrate of P-gp and the competition experiments combined with molecular docking analysis were also designed to clarify the binding sites of emodin on P-gp.

## Materials and methods

### Chemicals and drugs

Verapamil, adriamycin, cyclosporine A, rhodamine 6G, chrysophanol and rhodamine123 were purchased from Biosharp (Nanjing, China). Emodin and 3-[4, 5-dimethyl thiazol-2-y]-2, 5-diphenyl terazolium bromide (MTT) were bought from Sigma-Aldrich (St. Louis, USA). The BCA Protein Assay kit and RIPA lysis buffer were obtained from Beyotime (Beijing, China). Enhanced chemiluminescence (ECL) detection kit was from Advansta (Menlo Park, California, USA). The Annexin V Apoptosis Detection Kit APC and DAPI were available from eBioscience (San Diego, CA). The Lipofectamine RNAiMAX Reagent was purchased from Invitrogen Trading Co., Ltd (Shanghai, China).

### Cell lines and cell culture

Human myelogenous myeloid leukemia (K562) cells and the P-gp overexpressing adriamycin-resistant K562 cells (K562/ADM) were obtained from Institute of Hematology of Chinese Academy of Medical Sciences (Tianjin, China). All CML cell lines were cultured in RPMI 1640 media (Thermo Fisher Scientific,Waltham, MA, USA) supplemented with 10% fetal bovine serum (FBS) (Gibco BRL, Grand Island, NY, USA),100 U/ml of penicillin and 100 μg/ml of streptomycin (Sigma, St. Louis, Mo, USA) at 37°C in a humidified atmosphere of 5% CO_2_. K562/ADM cells were cultured in the presence of 5 μg/ml adriamycin. Before the experiments, adriamycin was withdrawn from the cells for two weeks.

The Caco-2 cell line (ATCC #HTB-37) was purchased from the American Type Culture Collection. The Caco-2 cells were grown in Dulbecco's modified Eagle's medium (DMEM) (Thermo Fisher Scientific, Waltham, MA, USA)supplemented with 10% FBS, 100 U/ml of penicillin and 100 μg/ml of streptomycin at 37°C in a humidified atmosphere of 5% CO_2_.

### Cell cytotoxicity assay

To determine the dose-dependent and time-dependent cytotoxicity of emodin, 6×10^4^ cells/well of K562 and K562/ADM (cells grown in suspension) were seeded in 96-well plates. The cytotoxicity was assessed using the conventional MTT assay as reported with minor modification [13]. The emodin incubation time is 24, 48 or 72 h. Cell viability was calculated according to the formula: viability% = (OD emodin treated cells − OD emodin medium control)/(OD untreated cells − OD medium control)× 100%

For Caco-2 (cells grown in adherence), 2×10^4^ cells/well were seeded in 96-well plates and incubated for 24 h, then the MTT assay was carried out as described above.

### Assay of the reversal efficacy

The potential of emodin to reverse MDR was evaluated by the MTT method in the K562/ADM cell line. K562/ADM cells were seeded into 96-well plates (6 ×10^4^ cells each well). Subsequently, adriamycin only or combinations with emodin (1–40 μM) or verapamil (10μM) diluted in RPMI1640 were added. The cells were maintained at 37°C in a humidified atmosphere of 5% CO_2_ for 48 h, and the percentages of viable cells were evaluated by the MTT assay and plotted against concentrations of the emodin to determine their half maximal inhibitory concentration (IC_50_). The reversal fold (RF) values were calculated according to the following formula: RF = IC_50_ of adriamycin alone/ IC_50_ of adriamycin combined with emodin. Each assay was repeated thrice.

### Apoptosis assay

K562/ADM cells (1×10^6^ cells/ml) were seeded in 6-well plates and treated with designated doses of adriamycin, emodin or verapamil for 72 h. The cells were double-labeled with Annexin V-APC and DAPI according to the manufacturer’s procedure. The cells were detected in 2 h on BD FACScantoII flow cytometer (BD Biosciences, San Jose, CA). The data were analyzed by FlowJo 7.6.2 software (Tree Star Inc., Ashland, OR). Triplicate experiments were implemented for flow cytometry analysis.

### Western blot analysis

P-gp antibody (sc-8313), secondary antibody (sc-2004), β-actin (sc-47778) were purchased from Santa Cruz Biotechnology, Inc. (Santa Cruz, CA). After treatment with emodin and adriamycin or siRNA transfection, cells were subjected to immuno-blot analysis and proceeded as previously reported [14]..

### siRNA transfection

K562/ADM cells (5×10^5^ cells/ml, 400 μl) were seeded in 24-well plates and transiently transfected with siRNA against P-gp (5’-GCGAAGCAGUGGUUCAGGUTT-3’) for 48 h at the final concentration of 100 nM using Lipofectamine RNAiMAX Reagent according to the manufacturer’s protocol. The negative control (5’-UUCUCCGAACGUGUCACGUTT-3’) in the transfection was used as a control siRNA. The protein expressions were detected by western blot analysis and cells with P-gp knock down were seeded for the emodin uptake assays.

### LC-MS /MS analysis

Liquid chromatography was performed on a Shimadzu LC-2010CHT series chromatographic system (Shimadzu, Nakagyo-ku, Kyoto, Japan) consisting of an on-line solvent degasser, a quaternary gradient pump, an auto-sampler and a column oven. Quantification was achieved with a Thermo-Finnigan TSQ Quantum Ultra AM LC–MS/MS system equipped with an electrospray ionization (ESI) source (Thermo-Finnigan, San Jose, CA, USA). Data acquisition was performed with Xcalibur 1.4 software (Thermo-Finnigan, San Jose, CA, USA). Chromatographic separation was performed on Agilent TC-C_18_ (5 μm, 150 mm × 4.6 mm) at 35°C. The mobile phases were water with 0.1% formic acid—acetonitrile (25:75, v/v) for emodin, ammonium formate—formic acid buffer (5 mM ammonium formate and 0.1% formic acid)-methanol (5:95, v/v) for rhodamine 123 at a flow rate of 1.0 ml/min. The auto-sampler was adjusted to 4°C for optimal stability. Negative ion detection mode was selected for emodin and positive one for rhodamine 123 both with the drying gas temperature of 350°C, an ion spray voltage of 3.5 kV at a flow-rate of 10 L/min. Quantification was performed by selected reaction monitoring (SRM) mode with precursor-product ion transitions: m/z 268.3 → 224.9 for emodin, m/z 252.9 → 224.9 for chrysophanol (internal standard); m/z 345.2 → 285.2 and 443.3 → 415.2 for rhodamine123 and rhodamine 6G (internal standard) with the collision energies of 44 eV and 35 eV respectively.

### Intracellular accumulation of rhodamine 123

The effect of emodin on the intracellular accumulation of rhodamine 123 was determined in Caco-2 cells and P-gp overexpressing K562/ADM cells. Generally, the cells treated with various concentrations of emodin (Caco-2, 10–200 μM; K562/ADM, 0.25–20 μM) and rhodamine 123 (3 μM) were incubated at 37°C for 120 min. P-gp inhibitor verapamil (200 μM) was also used as a positive control. Then cells were washed in PBS and lysed in 100 μl of 8 M carbamide solution by sonication. After high speed centrifugation, the cell lysate was spiked with 10 μl internal standard (rhodamine 6G) and 50 μl acetonitrile. After fully vortex-mixed, the mixture was centrifuged at 12,000 rpm for 10 min, and the supernatant was analyzed by LC-MS/MS. Cell protein was quantitated by BCA Protein Assay Kit.

### Intracellular uptake of emodin

To further evaluate whether emodin was the substrate of P-gp, the intracellular uptake of emodin was determined in K562 and K562/ADM cells under different condition where P-gp activity was regulated. In detail, the K562 and K562/ADM cells with or without P-gp knockdown were treated with 5 μM emodin alone or combined with classic P-gp inhibitors (verapamil and cyclosporin A) and incubated at 37 or 4°C for 120 min. The cells were collected and the intracellular amount of emodin was determined by LC-MS/MS as described above. Cell protein was quantitated by BCA Protein Assay Kit.

### Interaction between emodin and rhodamine 123 or verapamil

To investigate the interaction between emodin and rhodamine 123, K562/ADM cells were coadministrated with different concentrations of emodin (0.25, 0.5, 1, 2.5, 5, 20 μM) with required concentration of rhodamine 123 (1, 3, 10 μM) for 120 min at 37°C. Similarly, to investigate the interaction between emodin and verapamil, 20 μl rhodamine 123 was added to K562/ADM cells to give a final concentration of 1 μM, and different concentrations of emodin (0.25, 0.5, 1, 2.5, 5, 20 μM) with required concentration of verapamil (0.5, 1, 2 μM) were coadministrated for 120 min at 37°C. The intracellular concentrations of rhodamine 123 were determined by LC-MS/MS method described above. Cell protein was quantitated by BCA Protein Assay Kit.

### Calculation of *K*_*intrinsic*_ (*K*_*i*_)

The value of *K*_*i*_ is the derived value of the Michaelis parameter standing for the effectiveness of chemosensitiszers on counteracting the effect of P-gp on the accumulation of the substrates of P-gp [6]. The value of *K*_*i*_ was calculated by equation:

$$D_{i}=D_{0}+\left(D_{\infty }-D_{0}\right)\times C/(K_{i}\times D_{\infty }/D_{0}+C),$$

where *D*_*i*_, is the intracellular amount of probe (μg/mg protein) when the concentration of inhibitor was *C* (μg/ml). *D*_*∞*_ and *D*_*0*_ represent intracellular amount of probe when the concentration of the inhibitors is infinity and infinitesimal. The value of *K*_*i*_ increases when two chemosensitizers coadministrated to inhibit P-gp compete with each other while if noncompetition exists, the value of *K*_*i*_ would remain unchanged [15].

### Docking studies

The binding modes of emodin with P-gp were further evaluated by molecular docking analysis, which was made with a refined murine P-gp derived from the original crystallographic data (PDB: 3G60), comprising 87% overall sequence identity and nearly 100% identity within the DBP with the exception of mSer725/hAla729 between mouse and human P-gp [16]. The molecular docking analysis was conducted as previously reported with minor modification [17]. In MOE, the R- and M- binding sites of P-gp were defined according to the amino residues mapped by Ferreria et al [18].

### Statistical analysis

All experiments were performed in triplicate. Data were presented as mean ± S.D. To test statistically significant differences among multiple treatments for a given parameter, one-way analysis of variance (ANOVA) was performed. A *p*-value smaller than 0.05 was considered as significant.

## Results

### Cytotoxicity of emodin

**Fig 2:**
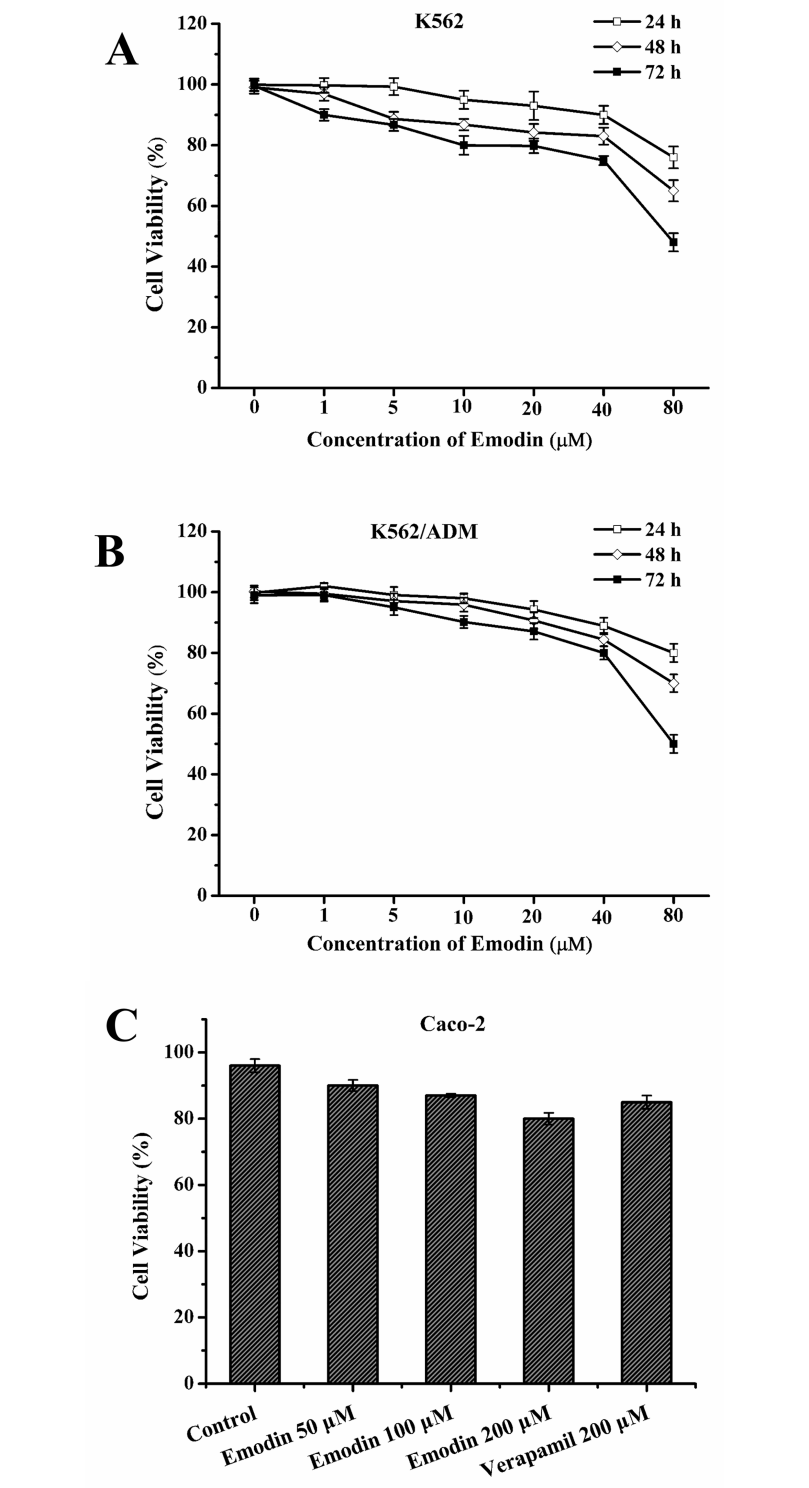
Effect of emodin on the viability of K562, K562/ADM cells and Caco-2 cells. The K562 (A), K562 /ADM (B) cells were treated with emodin at different doses for 24, 48 or 72 h, Caco-2 (C) cells for 48 h respectively. Data were expressed as means ± standard deviations (S.D.) of three independent experiments.

To choose appropriate doses of emodin to add into the cells, the cytotoxic activity of emodin on K562, K562/ADM and Caco-2 cells was evaluated by MTT assay. Emodin inhibited the viability of human leukemia K562 cells and its adriamycin-resistant K562/ADM cells in a dose-dependent and time-dependent manner (Fig 2A and 2B). Emodin of concentrations under 20 μM had a very weak cytotoxicity (cell viability>80%) to K562 and K562/ADM cells. To minimize the effect of emodin itself on K562 and K562/ADM cells growth, emodin concentrations from 0 to 20 μM were selected for the following experiments. In Caco-2 cells, emodin of 50, 100 and 200 μM and verapamil of 200 μM showed no significant cytotoxicity (Fig 2C) and these concentrations were selected for the following experiments.

### Emodin enhanced the sensitivity of K562/ADM cells to adriamycin

The reversing effect of emodin on adriamycin resistance was tested in K562/ADM cells. Verapamil, a well-known P-gp inhibitor, was used as a positive control. As shown in Table 1, emodin enhanced the sensitivity of K562/ADM cells to adriamycin by 1.60, 2.47, 2.96, 4.91, 9.30-fold at the concentrations of 1, 5, 10, 20, 40 μM, respectively, in a dose-dependent manner. The RF value of 10 μM verapamil was 2.66, which was comparable to that of emodin in the same dose, hinting the similar MDR-reversal activity between emodin and verapamil.

**Table 1:** Effect of emodin on adriamycin cytotoxicity in K562/ADM cells.

Treatment	K562/ADM	
	IC_50_ (μM)	RF
Adriamycin	10.97	-
Adriamycin + 1 μM Emodin	6.34 ± 0.12**	1.6
Adriamycin + 5 μM Emodin	4.01 ± 0.15**	2.47
Adriamycin + 10 μM Emodin	3.20 ± 0.22**	2.96
Adriamycin + 20 μM Emodin	1.53 ± 0.28**	4.91
Adriamycin + 40 μM Emodin	0.99 ± 0.17**	9.30
Adriamycin + 10 μM Verapamil	3.05 ± 0.25**	2.66

Effect of emodin on the sensitivity of K562/ADM cells toward adriamycin were examined by MTT method as described above. The cells were treated with various concentrations of adriamycin in the presence of emodin for 48 h. The IC_50_ values for adriamycin were calculated. The results were presented as means ± S.D. from three independent experiments.

*p< 0.05,

** *P*<0.01. RF = IC_50_ of adriamycin / IC_50_ of adriamycin and emodin in combination.

**Fig 3:**
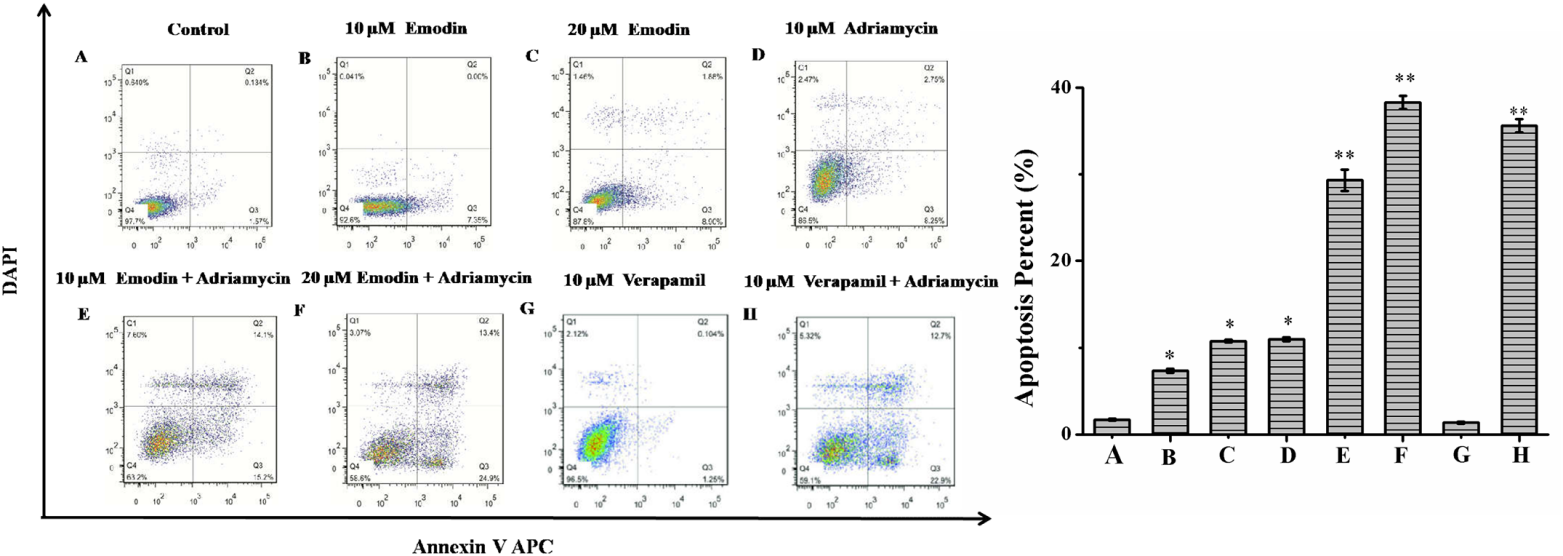
Effect of emodin on adriamycin-induced apoptosis in K562/ADM cells measured by annexin V-APC/DAPI double-staining assay. The K562/ADM cells were treated with adriamycin (10 μM), emodin (10 and 20 μM) or verapamil (10 μM) alone;, or adriamycin (10 μM) combined with emodin (10 and 20 μM) or verapamil (10 μM) for 48 h. Then cells were stained with annexin V-APC and DAPI before being subjected to flow cytometry for analysis. Histogram represented the means ± S.D. values for apoptotic cells obtained from three independent experiments. *P < 0.05; ** *P* < 0.01.

To further ascertain the effect of emodin on the adriamycin-induced cytotoxicity in K562/ADM cells, an apoptosis assay was executed in K562/ADM cells treated with adriamycin alone or adriamycin in combination with emodin. Considering the maximal fluorescence spectrum of emodin was between 515 nm and 525 nm, cells were double-stained with annexin V-APC and DAPI in order to avoid fluorescence interference of emodin [19, 20]. As shown in Fig 3, compared with the control group, K562/ADM cells treated with 10, 20 μM emodin or 10 μM adriamycin showed apparent apoptosis with cell apoptosis rates of 7.35%, 10.78% and 11%, respectively. In contrast, treatment of 10 or 20 μM emodin or 10 μM verapamil combined with 10 μM adriamycin brought about a clearly increased cells number in the right areas with the apoptosis percents of 29.3%, 38.3%, 35.6%, respectively (*p*< 0.01). Therefore, it was demonstrated that emodin could enhance adriamycin-induced apoptosis in K562/ADM cells in a dose-dependent manner with similar effect of verapamil.

### Emodin suppressed expression of P-gp protein in K562/ADM cells

**Fig 4:**
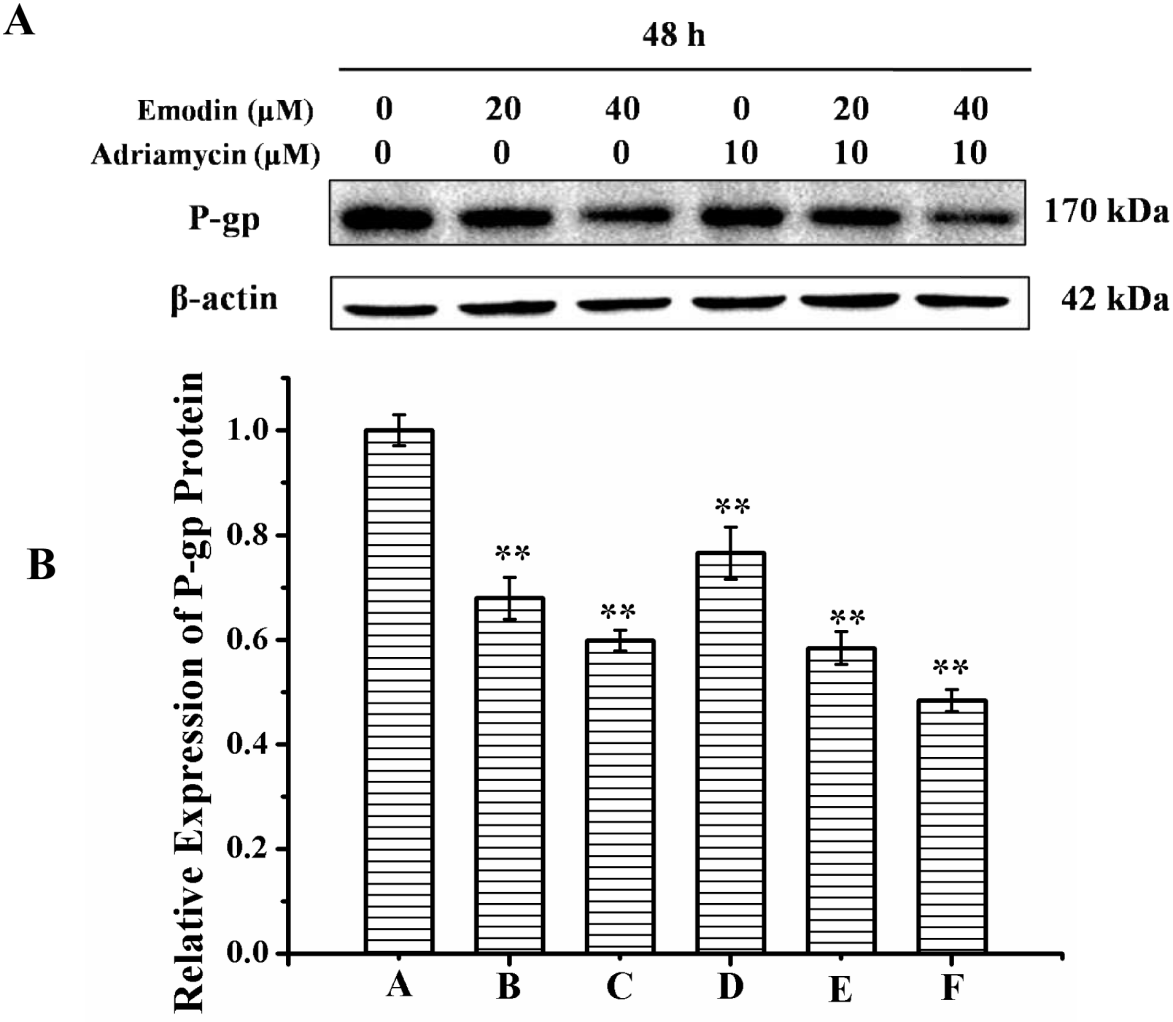
Effect of emodin on expression of P-gp protein in K562/ADM cells. The cells were incubated in absence or presence of adriamycin or emodin for 48 h. The protein expression was determined by western blot analysis (A) and the relative expression of P-gp was calculated (B). *p< 0.05; **p< 0.01.

Since the reversal of P-gp mediated MDR might be associated with the alteration of protein expression, emodin was examined for the potential to modulate the P-gp protein expression in K562/ADM cells. As illustrated in Fig 4A and 4B, the treatment of 20 or 40 μM emodin alone or combined with 10 μM adriamycin exerted significant downregulation of P-gp protein expression compared with the control group and the group treated with 10 μM adriamycin alone. These results suggested that emodin suppressed P-gp protein expression in human K562/ADM cells, which might contribute to its MDR reversal activity.

### Emodin increased intracellular accumulation of rhodamine 123

**Fig 5:**
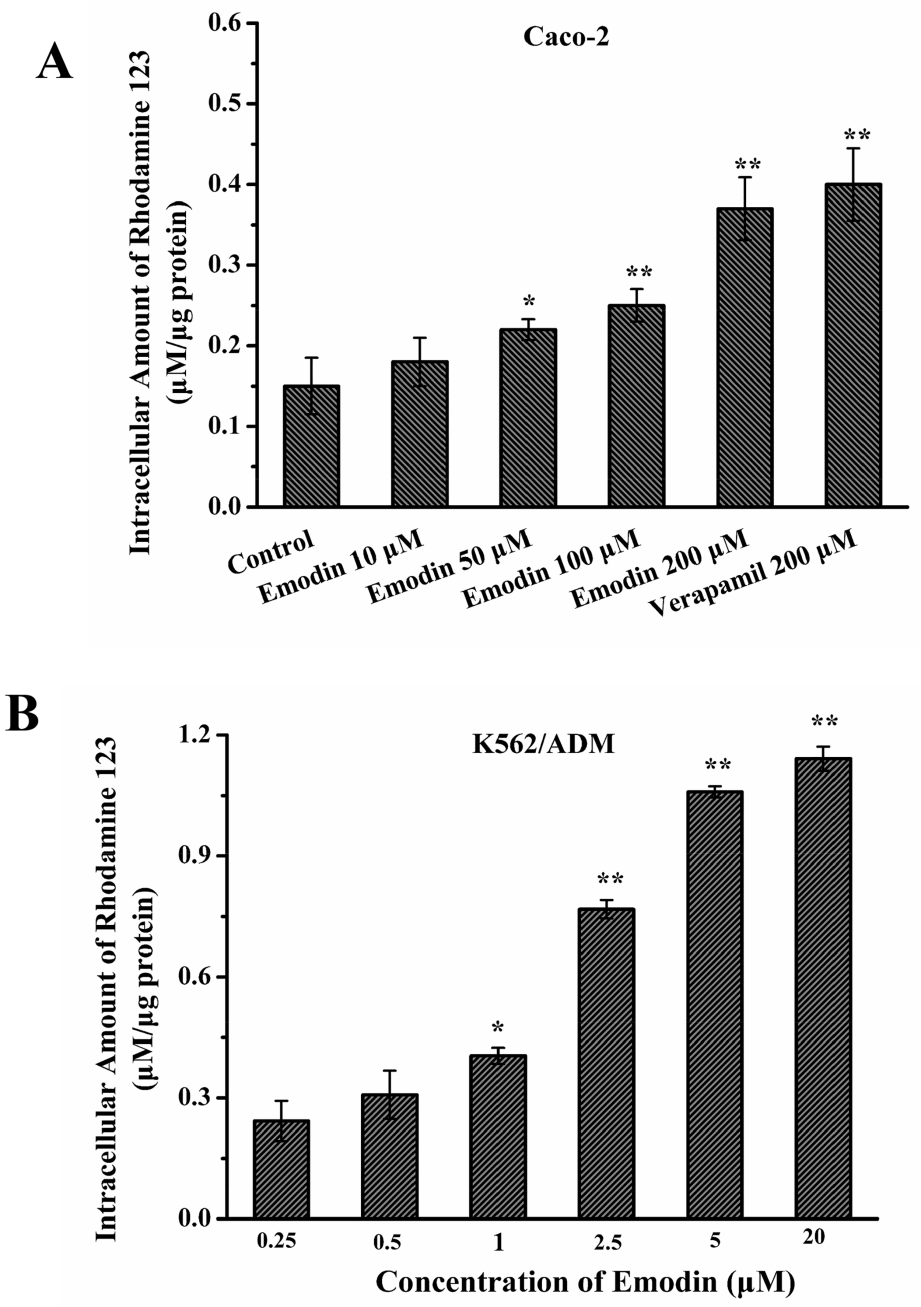
Effects of emodin on rhodamine 123 uptake in Caco-2 and K562/ADM cells. The concentration-dependent effects of emodin on rhodamine 123 accumulation in Caco-2 (A) and K562/ADM (B) cells were investigated, respectively at final concentrations of 10, 50, 100, 200 μM; 0.25, 0.5, 1, 2.5, 5, 20 μM. Data were represented as the mean ± S.D. of three independent experiments. *p < 0.05; ** *p*< 0.01.

The decrease of intracellular drug concentrations, a result of the efflux of anticancer drugs in tumor cells, is believed to be a common cause of P-gp-mediated MDR. To investigate whether emodin could inhibit the efflux function of P-gp, the intracellular accumulation of rhodamine 123, a classical substrate of P-gp, was measured in the Caco-2 and K562/ADM cells by LC-MS/MS. As shown in Fig 5, emodin concentration dependently increased the intracellular accumulation of rhodamine 123 in Caco-2 and K562/ADM cells. In Caco-2 cells, emodin exhibited a similar efficiency on the rhodamine 123 accumulation at 20 μM compared with 200 μM verapamil (Fig 5A). These data demonstrated that emodin could inhibit the P-gp efflux function to modulate MDR in K562/ADM cells as well as in Caco-2 cells.

### Emodin inhibited P-gp function as a substrate

**Fig 6:**
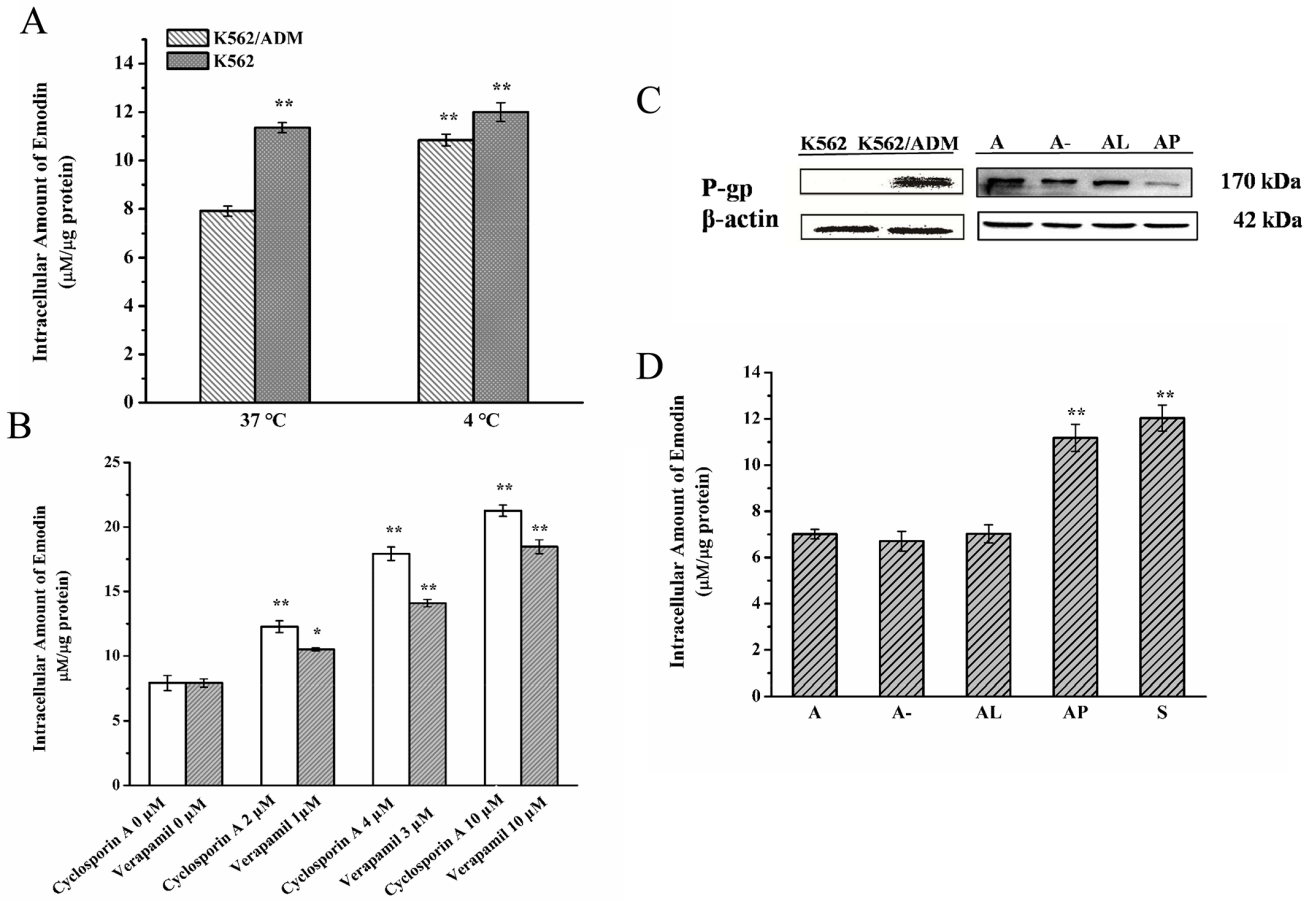
Intracellular amount of emodin in K562/ADM and K562 cells. Both cells were incubated with various concentrations of emodin (0.1–10 μM) at 37°C or 4°C for 2 h (A); K562/ADM cells were co-treated with 0, 1, 3, 10 μM of verapamil or 0, 2, 4, 10 μM of cyclosporine A and emodin (5 μM) for 2 h (B). RNAi of P-gp (C). A-: K562/ADM cells transfected with negative control siRNA; AL: K562/ADM cells transfected with Lipofectamine RNAiMAX Reagent; AP: K562/ADM cells transfected with P-gp siRNA; A: K562/ADM cells; S: K562 cells. After transfection with P-gp siRNA, intracellular amount of emodin (5 μM) was enhanced compared with other control groups (D). Data were represented as the mean ± S.D. of three independent experiments. *P < 0.05; ** *P* < 0.01.

To clarify whether emodin is a substrate of P-gp, the intracellular amount of emodin was determined by LC-MS/MS in sensitive K562 and resistant K562/ADM cells under different conditions where the P-gp function were inhibited. The data showed the amount of emodin in K562 cells was higher than that in the K562/ADM cells at 37°C (*p*< 0.01, Fig 6A). In addition, compared with K562/ADM incubated at 37°C, K562/ADM cells incubated at 4°C, at which the P-gp efflux activity was inactivated, showed higher intracellular emodin concentrations (*p*< 0.01). On the contrary, insensitive K562 cells, the intracellular amount of emodin was nearly at a similar level in K562 cells incubated at 37°C and K562/ADM incubated at 4°C (Fig 6A). Furthermore, two classic P-gp inhibitors, verapamil and cyclosporine A could effectively increase the accumulation of emodin in K562/ADM cells (Fig 6B). Besides, the RNAi technology was also introduced to down-regulate the P-gp protein level in K562/ADM cells successfully (Fig 6C). Obviously, the concentration of emodin was much higher in the P-gp knockdown K562/ADM cells compared with that of the controls, while comparable with that in K562 cells (*p* < 0.01, Fig 6D). All the above results provided reliable evidence that emodin was most likely the substrate of P-gp and it could be effuxed by P-gp.

### Emodin and rhodamine 123 shared the common binding sites in K562/ADM cells

**Fig 7:**
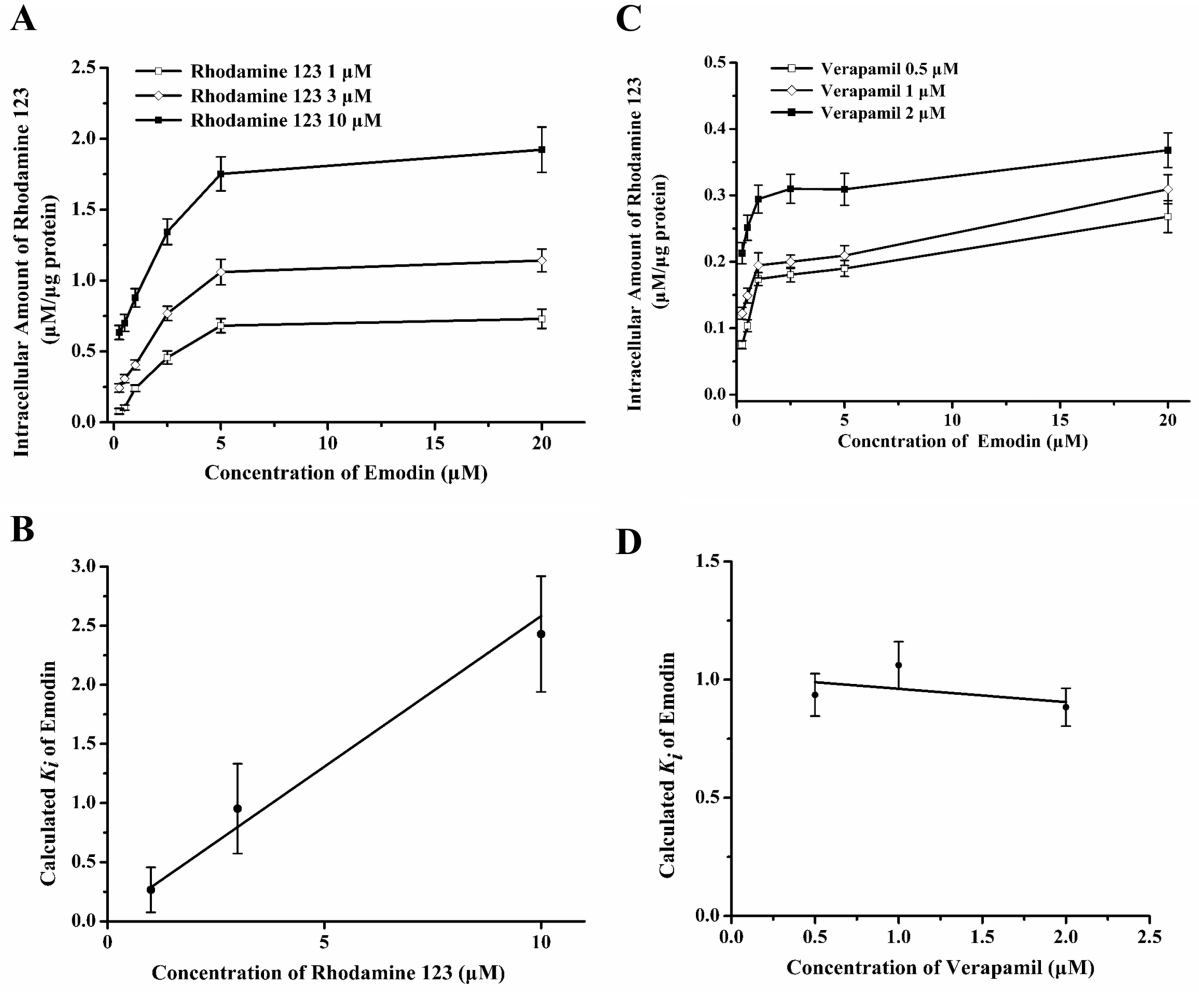
Effect of emodin combined with various amount of rhodamine 123 or verapamil on inhibition of P-gp. K562/ADM cells were incubated with emodin (0.25–20 μM), together with 1, 3, 10 μM of rhodamine 123, respectively (A); The *K*_*i*_ of emodin was calculated and plotted against the concentrations of rhodamine 123 (B). K562/ADM cells were incubated with emodin (0.25–20 μM) with 0.5, 1, 2 μM of verapamil and 1 μM of rhodamine 123 (C). The calculated values of *K*_*i*_ for emodin were plotted against the concentrations of verapamil (D). Data were represented as the mean ± S.D of three independent experiments.

Previous studies confirmed that the classic substrate of P-gp, rhodamine 123, acts with P-gp on the binding site called R [21 22]. Since the present study showed that emodin was a substrate of P-gp, the competition style between emodin and rhodamine 123 was investigated to see whether the two substrates shared the same binding sites when pumped out by P-gp. The three curves in Fig 7A showed the increase of rhodamine 123 accumulation brought about by increasing variable substrate emodin at the range of 0 to 20 μM, while the fixed substrate rhodamine 123 was set at 1, 3 and 10 μM. From each curve, the values of *K*_*i*_ for emodin were computed and plotted against the rhodamine 123 concentrations. The *K*_*i*_ clearly increased consistently with the increasing rhodamine 123 concentrations (Fig 7B), indicating competition interactions between emodin and rhodamine 123. Thus, based on the data obtained, it could be concluded that emodin and rhodamine 123 may share the same R binding site as P-gp substrates.

### Emodin displayed noncompetition with verapamil

Apart from rhodamine 123 as a substrate probe, the classic inhibitor verapamil which binds to P-gp at the binding sites called the M site was also used as a fixed modulator to verify the binding sites of emodin on P-gp [18, 23]. The calculated *K*_*i*_ values were obtained for emodin and plotted against the verapamil concentrations. As shown in Fig 7D, the *K*_*i*_ of emodin was almost unchanged with increasing verapamil concentrations, confirming that emodin and verapamil were non-competitors or else very weakly competitive. Consequently, it is reasonable to conclude that verapamil increased the accumulation of emodin in a non-competition style at the M site (Fig 6B).

### Molecular docking analysis

**Fig 8:**
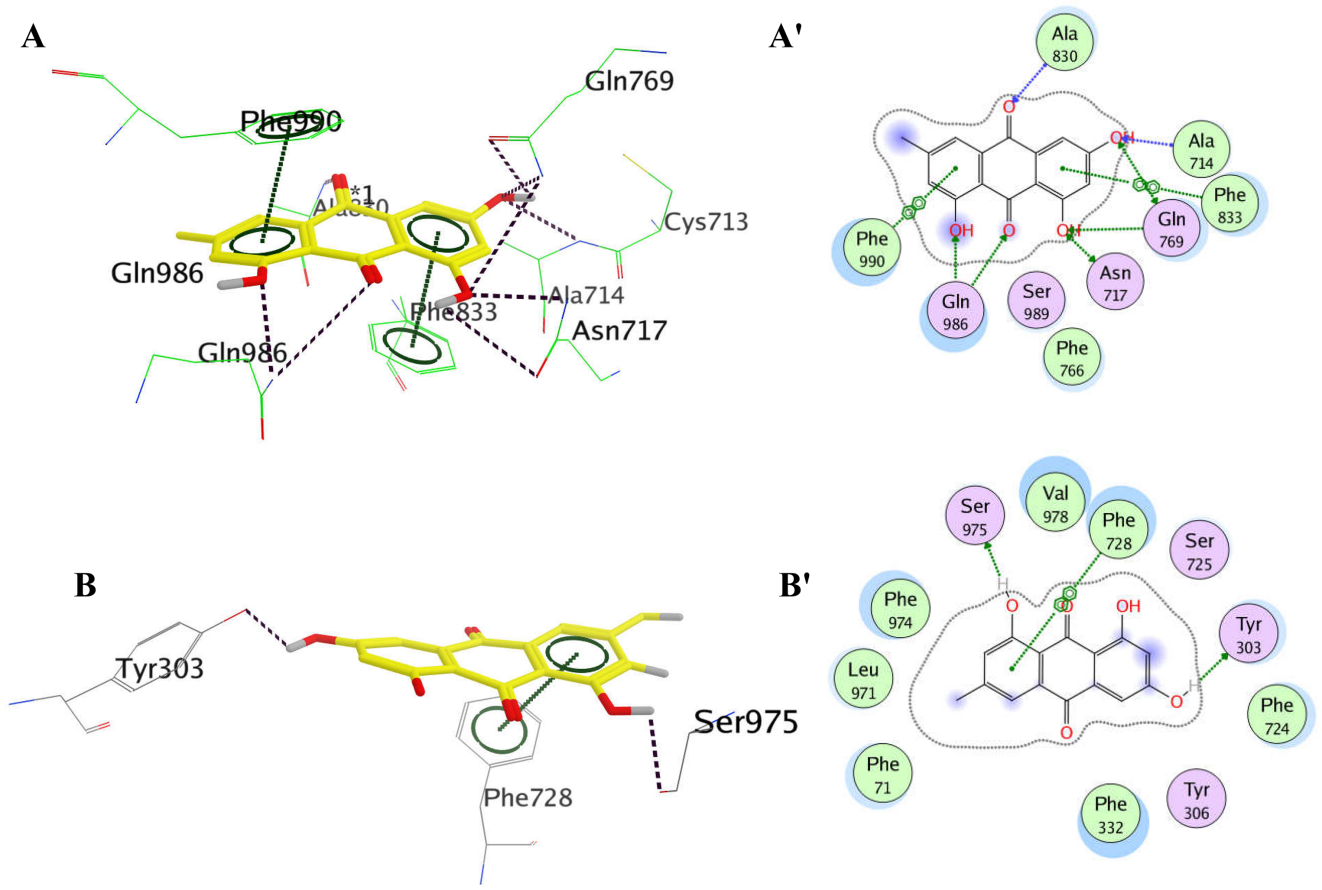
Docking views of emodin with P-gp. The three-dimensional diagrams show the interactions and preferred conformation of emodin on the R site (A) and the M site (B) with labeled amino residues which significantly contributed for binding free energy. Emodin is colored in yellow. The hydrogen-bond is represented in purple dotted line. The π-π interaction is represented in green dotted lines. The two-dimensional diagrams display the interactions of emodin to the amino acid residues at the emodin on the R site (A') and the M site (B') of P-gp.

To further verify the detailed modes of emodin interacting with P-gp on R- and M- binding sites, molecular docking was performed among emodin and the X-ray crystal structure of P-gp. According to the molecular docking results, emodin bound to the R binding site with the binding free energy of -28.612 kcal/mol (Table 2). As shown in Table 2, Fig 8A and 8A', at R binding site, emodin formed seven H-bonds with residues Ala 830, Ala714, Gln 769, Asn 717, Gln 986, and provided two strong π-π interactions with Phe 990 and Phe 833. In contrast, it bound to the M site with a less negative binding free energy of -21.437 kcal/mol (the less negative binding free energy of emodin with P-gp binding sites means the less easier interaction with P-gp) than that of R site and formed only one π-π interaction with Phe 728 as well as two H-bonds with Ser 975 and Tyr 303 at the M site (Table 2, Fig 8B and 8B'). Combined, emodin could bind to the R binding site of P-gp with a much higher affinity than to the M site.

**Table 2:** Free binding energies and residues identified to interact with emodin dependent on the binding sites.

Binding sites	Free binding energy (kJ/mol)	Amino residues
R site	-28.612	Ala 830, Ala714, Phe 833, Gln 769, Asn 717, Phe 766, Ser 989, Gln 986, Phe 990
M site	-21.437	Ser 975; Val 978; Phe 728; Ser 725; Tyr 303; Phe 724; Tyr 306; Phe 332; Phe 71; Leu 971; Phe 974,

## Discussion

Multidrug resistance (MDR) is an obstacle to successful cancer chemotherapy [24], one of the main mechanisms being the overexpression of P-gp. Similarly, P-gp mediated MDR in CML is also a key factor that leads to the failure of imatinib-based chemotherapy [25, 26]. K562/ADM, a P-gp-overexpressed adriamycin resistant CML cell line, shows resistance not only to adriamycin itself, but also to a broad range of anticancer reagents, including vincristine, etoposide and the one line CML chemotherapeutic drug imatinib [27, 28]. Consequently, the K562/ADM cell line was selected to evaluate the reversal effects of emodin on MDR in CML and to investigate whether emodin is the substrate of P-gp as well as its binding sites to P-gp.

In our study, emodin significantly reversed the adriamycin resistance in K562/ADM cells, which was also confirmed by the apoptosis assay. The results of western blot analysis revealed that emodin alone or combined with adriamycin concentration dependently decreased the P-gp protein level. Meanwhile, emodin increased the intracellular amount of rhodamine 123 in both Caco-2 and K562/ADM cells. In other words, emodin is an effective inhibitor of P-gp by indirect mechanisms related to the decrease of P-gp protein expression or directly binding to P-gp and then weakening the P-gp function, coincident with previous reports [9, 10, 29]. In addition, the LC-MS/MS method was established for the assay of rhodamine 123 and emodin in K562/ADM and Caco-2 cells owing to the strong fluorescence interference between emodin and rhodamine 123 as a result of the maximum emission wavelength of the two compounds both being around 525 nm [19, 30].

As for whether emodin was the substrate of P-gp, it was previously proposed that emodin was the potential substrate of P-gp [11]. Differently, Zhang et al reported that emodin was not the substrate of P-gp based on a one way intestinal perfusion rat model [12]. Herein, to clarify the discrepancy, many methods were applied to inhibit the function of P-gp. RNAi, one of the most powerful tool to suppress the protein expression, was introduced to down regulate the P-gp protein level in K562/ADM cells. Besides, at 4°C, the P-gp efflux function will be weaken [31, 32], thus the K562/ADM cells were incubated both at 37 and 4°C. Moreover, the classical P-gp inhibitors verapamil and cyclosporine A were also used. As shown, when the P-gp protein level was decreased or the P-gp effux function was inhibited, the intracellular amount of emodin remarkably increased, which proved that P-gp mediated the efflux of emodin in K562/ADM cells. In conclusion, consistent with Li et al [11], it was more comprehensively and reasonably recognized that emodin was a substrate for P-gp.

In successive years, many efforts were paid to elucidate the presence of multiple drug binding sites on P-gp. Safa et al have reported by kinetic analysis that vinblastine and cyclosporin A competitively interact competitively with a common binding site on P-gp whereas while they noncompetitively interact with the azidopine-binding site, which indicate there are more than one binding site of P-gp [33,34]. Later, a more complete view believed that three drug binding sites existed on P-gp [21]. More recently, after the the publication in 2009 of the murine crystallographic structure that clarified P-gp’s structural properties, three putative drug-binding sites were hereby characterized by means of molecular docking, two of which are named as R (Rhodamine) site and M (modulatory) site [18,23,35]. Previous studies indicated that rhodamine 123 and photoaffinity analogs of verapamil could both bind specifically to p-gp [36, 37]. Competition studies confirmed that rhodamine 123 bound to the R site preferentially [21], while molecular docking studies show that verapamil binds to the M site as a modulator [18]. The clarification of explicit binding sites of emodin on P-gp will provide vital information for chemical modification to develop emodin into an effective P-gp inhibitor, thereby, the investigation of the emodin binding sites is of important priority. Hence, in the present study, competition studies were performed between emodin and rhodamine 123 or verapamil for the first time. The *K*_*i*_ values of emodin were calculated according to the formula created by Stein’s group based on the classical Michaelis-Menten scheme, which successfully described the competitive, noncompetitive, and cooperative interactions between several P-gp modulators [15]. A competitor binds to the same site when it competes with other substrates and the value of *K*_*i*_ increased linearly with the concentration of other substrates, while if noncompetition exists, the *K*_*i*_ value will be unchanged [6, 15]. Meaningfully, the *K*_*i*_ of emodin increased linearly with the concentrations of rhodamine 123 while remained nearly unchanged with increasing verapamil concentrations, which indicated emodin competitively bound to P-gp with rhodamine 123 at the R site, defined as a transport site, whereas no or else very weak competition existed between verapamil and emodin. These results were consistent with previous studies that most modulators displayed their MDR reversal activities by competitively binding to the transport site [38]. Nevertheless, it was still uncertain whether emodin could bind to the M site.

Molecular docking analysis helped to provide much more information on the interaction modes of emodin with P-gp drug binding sites. According to amino residues mapped by Ferreia et al [18], the R- and M- sites of P-gp were defined as the docking cavity of emodin for the first time. Then, the binding free energies and the interactions of emodin at R- and M- sites were obtained to evaluate the binding affinity of emodin. Generally, the more negative binding free energy means the easier interaction with each other. The docking studies showed that emodin bound to the R site with a more negative binding free energy than to the M site and formed more Pi interactions and H bonds at R site, which were in reasonable agreement with the observed results of competition experiments that emodin competed with rhodamine 123 (R site) rather than verapamil (M site) at P-gp. Partially coincident with our results, it was reported by Li et al that emodin had four potential Pi interactions with Phe974 and Phe728 in the internal cavity of P-gp [11]. As to the differences between the docking studies, it had to be noted that our docking cavity was refined in the R- and M- sites at P-gp respectively while Li et al performed the docking study in the whole internal cavity of P-gp. In comparison, our docking studies provided more accurate binding modes of emodin at the R- and M- sites. Additionally, due to the lack of a high resolution crystal structure of human P-gp, all molecular docking analysis was based on the crystal structure of mouse P-gp [35] or different homology model structures of human P-glycoprotein [39–41], therefore it could be understood that in some cases the docking data showed a great amount of controversy. All in all, the binding sits of emodin on P-gp needs further confirmation in future studies.

## Conclusion

In summary, the present study verified that emodin reversed P-gp mediated MDR in K562/ ADM cells through the inhibition of P-gp protein expression and its drug efflux function as a substrate. Our data firstly provided direct evidence that there is competitive interaction among emodin and rhodamine 123 at the R site of P-gp, whereas emodin noncompetitively bound to the M site with verapamil. Taken together, we suggest that emodin might be used to modulate P-gp function and expression. In future, more detailed pharmacokinetic and pharmacodynamics studies are needed to reveal the underlying molecular mechanisms of emodin in the reversal of P-gp mediated MDR.

## Abbreviations

ABC: ATP-binding cassette
APC: Allophycocyanin
CML: chronic myelogenous leukemia
DAPI: 2-(4-Amidinophenyl)-6-indolecarbamidine dihydrochloride
DMEM: Dulbecco's modified Eagle's medium
ECL: enhanced chemiluminescence
FBS: fetal bovine serum
IC_50_: half maximal inhibitory concentration
K_i_: *K*_*intrinsic*_
MDR: multidrug resistance
MDR1: multiple drug resistance 1
MTT: 3-(4, 5-dimethylthiazol-2-yl)-5-(3-carboxymethoxyphenyl)-2-(4-sulfophenyl)-2H-tetrazolium
PBS: phosphate buffer solution
P-gp: P-glycoprotein
PVDF: polyvinylidine difluoride
RF: reversal fold
RNAi: RNA interference
RPMI 1640: Roswell Park Memorial Institute 1640 medium
TKIs: tyrosine kinase inhibitors
